# Comparative cytogenetics of the ground frogs *Eupsophus emiliopugini* Formas, 1989 and *E. vertebralis* Grandison, 1961 (Alsodidae) with comments on their inter- and intraspecific chromosome differentiation

**DOI:** 10.3897/CompCytogen.v14i1.46852

**Published:** 2020-01-27

**Authors:** Camila A. Quercia, Elkin Y. Suárez-Villota, Fausto Foresti, José J. Nuñez

**Affiliations:** 1 Instituto de Ciencias Marinas y Limnólogicas, Universidad Austral de Chile, Edificio Emilio Pugin, Campus Isla Teja S/N, Casilla 567, Valdivia, Chile Universidad Austral de Chile Valdivia Chile; 2 Departamento de Morfologia, Instituto de Biociências, Universidade Estadual Paulista, Distrito de Rubião Junior, s/n, 18618-970, Botucatu, São Paulo, Brazil Universidade Estadual Paulista Botucatu Brazil

**Keywords:** Karyotype variations, FISH, Patagonian frogs, ribosomal probe, NOR polymorphism

## Abstract

South American frogs of the genus *Eupsophus* Fitzinger, 1843 comprise 10 species. Two of them, *Eupsophus vertebralis* Grandison, 1961 and *E. emiliopugini* Formas, 1989 belong to the *Eupsophus vertebralis* group, exhibiting 2n = 28. Fundamental number differences between these species have been described using conventional chromosome staining of few specimens from only two localities. Here, classical techniques (Giemsa, C-banding, CMA_3_/DAPI banding, and Ag-NOR staining), and fluorescence *in situ* hybridization (FISH, with telomeric and 28S ribosomal probes), were applied on individuals of both species collected from 15 localities. We corroborate differences in fundamental numbers (FN) between *E. vertebralis* and *E. emiliopugini* through Giemsa staining and C-banding (FN = 54 and 56, respectively). No interstitial fluorescent signals, but clearly stained telomeric regions were detected by FISH using telomeric probe over spreads from both species. FISH with 28S rDNA probes and Ag-NOR staining confirmed the active nucleolus organizer regions signal on pair 5 for both species. Nevertheless, one *E. emiliopugini* individual from the Puyehue locality exhibited 28S ribosomal signals on pairs 4 and 5. Interestingly, only one chromosome of each pair showed Ag-NOR positive signals, showing a nucleolar dominance pattern. Chromosomal rearrangements, rRNA gene dosage control, mobile NORs elements, and/or species hybridization process could be involved in this interpopulation chromosomal variation.

## Introduction

*Eupsophus* Fitzinger, 1843 is a South American genus of frogs that currently comprises 10 species (Frost 2019, Suárez-Villota et al. 2018a), endemic from the temperate *Nothofagus* forests from Chile and Argentina (Formas 1978, Ibarra-Vidal et al. 2004). Based on ethologic (advertisement calls; Formas and Brieva 1994), morphometrics (Nuñez 2003), molecular (allozymes and DNA sequences; Formas et al. 1992, Blotto et al. 2013), and cytogenetic (Formas 1991, Veloso et al. 2005) analyses, this genus is divided into the *Eupsophus roseus* and the *Eupsophus vertebralis* groups.

The *E. roseus* group is composed of eight species: *E. calcaratus* (Günther, 1881), *E. contulmoensis* Ortiz, Ibarra-Vidal & Formas, 1989, *E. septentrionalis* Ibarra-Vidal, Ortiz, & Torres-Pérez, 2004, *E. nahuelbutensis* Ortiz & Ibarra-Vidal, 1992, *E. insularis* (Philippi, 1902), *E. migueli* Formas, 1978, *E. roseus* (Duméril & Bibron, 1841), and *E. altor* Nuñez, Rabanal & Formas, 2012 (Suárez-Villota et al. 2018a) exhibiting the same diploid number 2n = 30 with some species specific characteristics (*e.g*. fundamental number, sex chromosomes, secondary constriction location; Iturra and Veloso 1986, Veloso et al. 2005, Nuñez et al. 2012). On the other hand, the *E. vertebralis* group, composed of *E. vertebralis* Grandison, 1961 and *E. emiliopugini* Formas, 1989, exhibit 2n = 28, do not have sex chromosomes, and present a secondary constriction in pair 5 (Formas 1991). Moreover, the pair 13 is metacentric in *E. emiliopugini* and telocentric in *E. vertebralis*, differing in their fundamental number (FN = 56 and FN = 54, respectively).

Having in mind the hypothetical ancestrality of telocentric chromosomes in amphibians (Morescalchi 1980), Formas (1991) proposed two alternative hypotheses to explain the origin of the differences on the pair 13 in the *E. vertebralis* group. The first one is a pericentric inversion in a telocentric pair of *E. vertebralis*, which shifted the centromere to the metacentric position in *E. emiliopugini*. The second hypothesis is the addition of heterochromatic segments in the centromeric region of the telocentric pair in *E. vertebralis*, which leads to a metacentric pair in *E. emiliopugini*. Formas (1991) considered the first alternative as a reasonable hypothesis because telocentric and metacentric pairs 13 are the same size.

Although the hypothesis of Formas (1991) is well argued from the data, it should be considered with caution since the conclusions are obtained using only conventional stain and specimens from only two locations, preventing the findings from being extrapolated, and increasing the chance of assuming as true a false premise. Here we combined classical and molecular cytogenetic techniques to characterize the karyotypes of these species using samples from several localities. Thus, we analyzed at population level the nucleolus organizer regions (NORs) position using Ag-NOR banding and fluorescent *in situ* hybridization (FISH) with 28S rDNA probe. Using FISH with telomeric probe and CMA_3_/DAPI banding, we sought interstitial signals, which could suggest chromosomal rearrangements in both species. Our comparative cytogenetic analyses provide a detailed description of the *E. vertebralis* group karyotypes and their inter- and intraspecific chromosome differentiation.

## Methods

### Sample collection and cytological preparations

**Figure 1.**
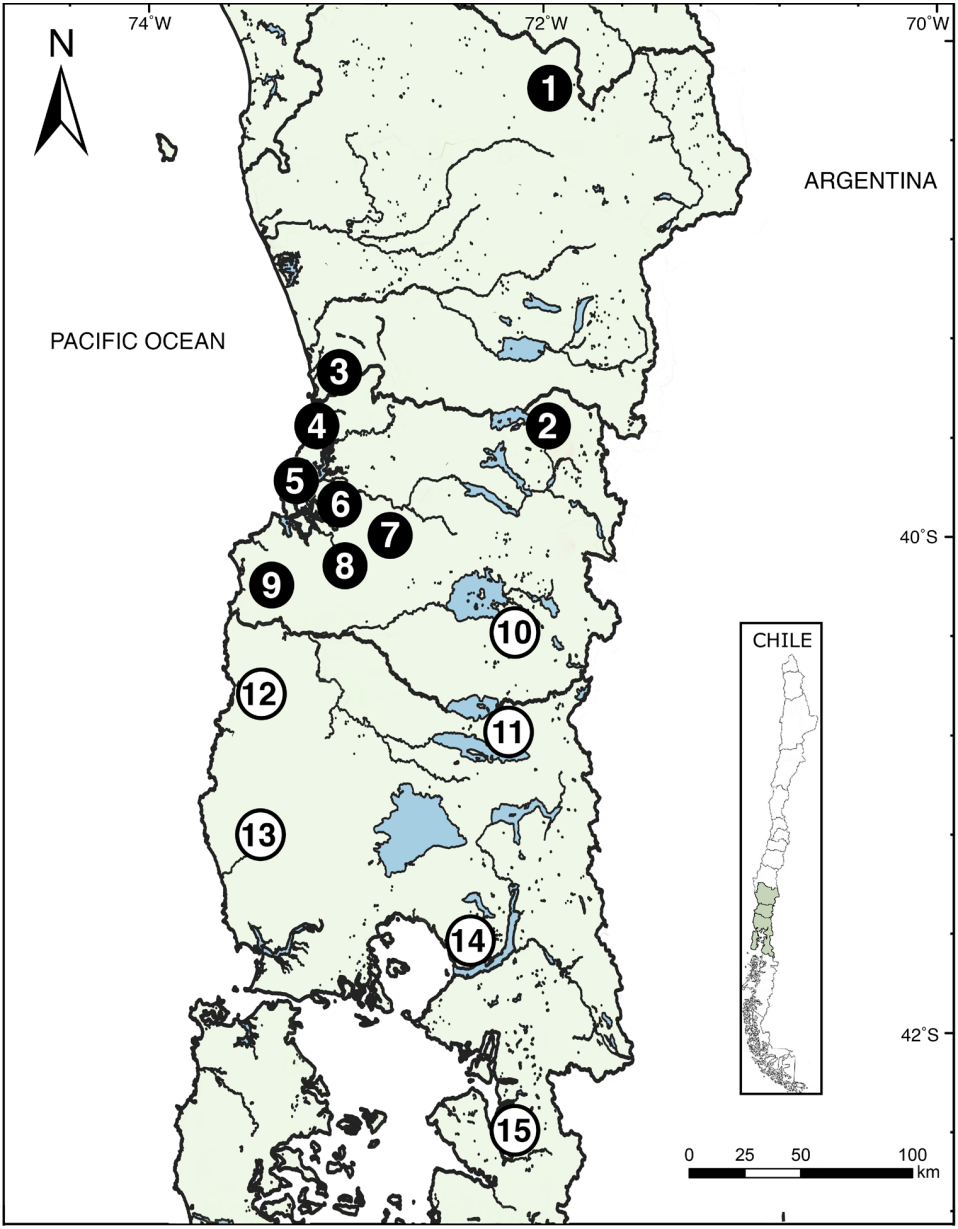
Map depicting 15 collection localities of the *Eupsophus vertebralis* group specimens in Southern Chile. *E. vertebralis* locations are represented by black circles, and *E. emiliopugini* locations are shown with white circles. The numbers inside the circles corresponds with the follow localities: **1**) Tolhuaca, **2**) Lago Pellaifa, **3**) Colegual Alto, **4**) Chanchan, **5**) Oncol, **6**) Llancahue, **7**) Reumén, **8**) Chamil, **9**) Cordillera Pelada, **10**) Los Mañios, **11**) Puyehue, **12**) Pucatrihue, **13**) Cordillera del Sarao, **14**) Parque Alerce Andino, and **15**) Huinay.

Cytological preparations were obtained from 14 and nine individuals of *Eupsophus vertebralis* and *E. emiliopugini*, respectively (See Suppl. material 1: Table S1). These individuals were collected according to permit of Servicio Agrícola y Ganadero (No. 9244/2015) from 15 locations in Southern Chile (Fig. 1). Mitotic plates were obtained from intestine cell suspension. For this purpose, we injected 30 µl/g of 0.1% colchicine (Sigma-Aldrich) into the abdominal cavity of each individual. After 12 hours, the individuals were euthanized with oversaturated benzocaine, according to the recommendations of the Bioethics and Biosecurity Committee of the Universidad Austral de Chile (UACh, resolution No. 236/2015 and 61/15). Immediately after euthanasia, the gut cells were extracted and prepared according to Schmid et al. (1978) protocol. Then, the specimens were included in the herpetological collection of Instituto de Ciencias Marinas y Limnológicas, UACh (voucher numbers in Suppl. material 1: Table S1).

### Classical cytogenetic techniques

Mitotic plates were stained with 10% Giemsa for karyotype determination. Active NORs were detected using silver nitrate staining (Ag-NOR) according to (Howell and Black 1980). This chromosomal material was analyzed in Siedentopf trinocular microscope (AmScope T340B-DK-LED) and photographed with AmScope camera using IS capture software. Karyotypes were arranged according to Formas (1991).

To identify constitutive heterochromatic regions, we carried out a C-banding protocol using formamide for DNA denaturation, according to Fernández et al. (2002) and staining with DAPI (1 μg/ml). CG-rich and AT-rich regions were detected using CMA_3_/DAPI stains, respectively follow to Schweizer (1976). In this technique, we used pretreated metaphases with formamide according to Pieczarka et al. (2006) as well as FISH pretreated plates (Suárez et al. 2013). For both C-banding and CMA3/DAPI stains, mitotic plates were mounted with Vectashield antifade. Subsequently, metaphases were visualized through a BX61 Olympus microscope, and captured with adequate filter using a DP70 Olympus digital camera with PRO MC Image software. All images were overlaid and contrast enhanced using Adobe Photoshop CS6.

### Molecular cytogenetic techniques

The physical map of the rDNA genes was detected by FISH on mitotic plates from *E. vertebralis* (from Colegual Alto and Reumén), and *E. emiliopugini* (from Puyehue, Cordillera del Sarao, and Parque Alerce Andino) specimens. For this purpose, 28S rDNA fragment from *E. vertebralis* DNA was amplified using 28SV (5´-AAGGTAGCCAAATGCCTCGTCATC-3´) and 28SJJ (5´-AGTAGGGTAAAACTAACCT-3´) primers (Hillis and Dixon 1991). PCR was carried out according to the manufacturer’s instructions for *Taq* Platinum DNA Polymerase (Cat. No. 10966, Invitrogen), at 55 °C of annealing temperature. The 28S probe was PCR-labeled with 11-digoxigenin dUTP (Cat. No. 11093088910, Sigma-Aldrich), hybridized according to Ferreira et al. (2011), and detected with Anti-Digoxigenin-Rhodamine, Fab fragments (Cat. No. 11207750910, Roche).

Telomere detection by FISH was carried out on metaphase chromosomes from *E. vertebralis* (from Tolhuaca, Reumén, and Colegual Alto), and *E. emiliopugini* (from Puyehue, Parque Alerce Andino, and Cordillera del Sarao) specimens. Universal telomeric probes (TTAGGG)_n_ were PCR-generated and labeled with fluorescein-12-dUTP (Cat. No 11373242910, Roche) (Ijdo et al. 1991). Fluorescent *in situ* hybridization followed to Ferreira et al. (2011) without final immunodetection protocol.

Slide mounting and image capture for both 28S rDNA and telomeric FISH assays were carried out as described previously for C-banding protocol.

## Results

### Classical cytogenetic techniques

**Figure 2.**
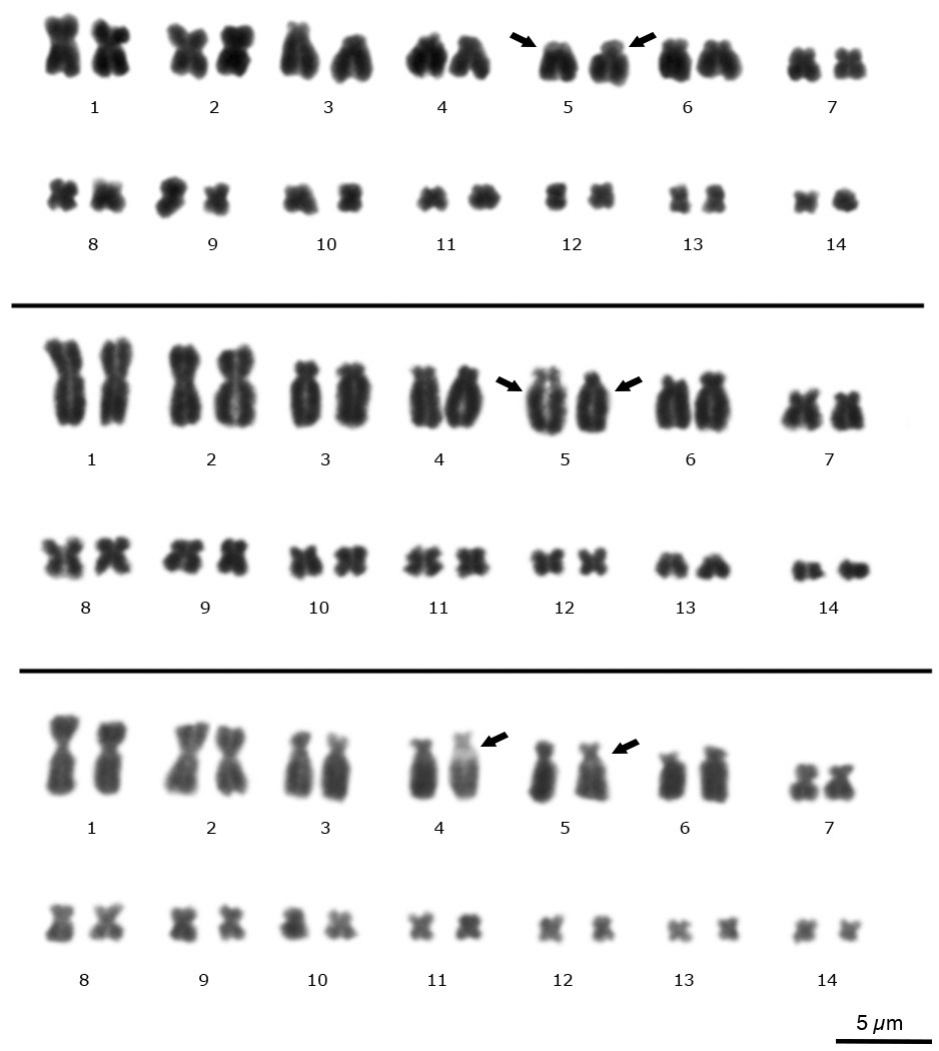
Conventional Giemsa banding on the *Eupsophus vertebralis* group mitotic plates. The *E. emiliopugini*, *E. vertebralis* and *E. emiliopugini* from Puyehue locality karyotypes are shown (top, middle, and bottom, respectively). Note metacentric (top and bottom) or telocentric (middle) pair 13. Secondary constrictions are indicated with black arrows on pairs 4 or 5 (see text for details).

We analyzed 88 mitotic plates showing 2n = 28 for each species, without evidence of sexual chromosomes (Fig. 2). All the *E. emiliopugini* plates showed only chromosomes bi-armed with a FN = 56. The pairs 1, 3, 8–14 were metacentric, pair 7 was submetacentric, and pairs 2, 4–6 were subtelocentric (Fig. 2, top) following the descriptions by Formas (1991).

Mitotic plates of *E. vertebralis* exhibited a telocentric pair 13 presenting FN = 54, while the other karyotypic features were similar to *E. emiliopugini* (Fig. 2, middle). Although it is not clear for all plates, secondary constriction was observed in the short arms of pair 5 from both species (Fig. 2, top and middle, black arrows). In one *E. emiliopugini* specimen collected at Puyehue (hereafter, the Puyehue specimen) was difficult to establish morphological homology among chromosomes of pairs 5 and 4 (Fig. 2, bottom, black arrows).

**Figure 3.**
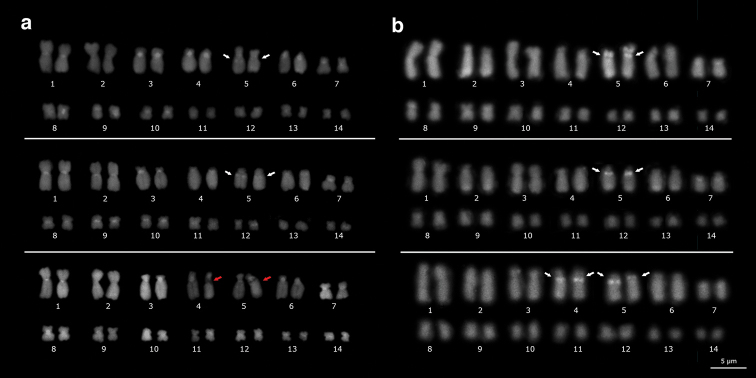
DAPI staining (**a**) and CMA_3_ (**b**) on the *Eupsophus vertebralis* group mitotic plates. The *E. emiliopugini*, *E. vertebralis* and *E. emiliopugini* from Puyehue locality karyotypes are shown (top, middle, and bottom, respectively). White arrows indicated heterochromatic interstitial bands in **(a)** and CMA_3_ positive signals in (**b**). Red arrows indicated secondary constriction in *E. emiliopugini* Puyehue specimen.

C-banding and DAPI staining detected predominantly centromeric regions in chromosomes of *E. emiliopugini* and *E. vertebralis* (Fig. 3a, top and middle, respectively). Interstitial heterochromatic signals were detected on the long arms of chromosomes of pair 5 (Fig. 3a, white arrows). When applying C-banding over mitotic plates from Puyehue specimen, secondary constrictions were detected in one chromosome of the pair 4, and in one chromosome of the pair 5 (Fig. 3a, bottom, red arrows). This final arrangement among chromosomes of pairs 4 and 5 was based on Ag-NOR technique as described below. CMA_3_ positive signals were detected on pair 5 of both karyotypes (Fig. 3b, top and middle, white arrows), but in that of the Puyehue specimen, these signals were detected in both chromosomes on pairs 4 and 5 (Fig. 3b, bottom, white arrows).

**Figure 4.**
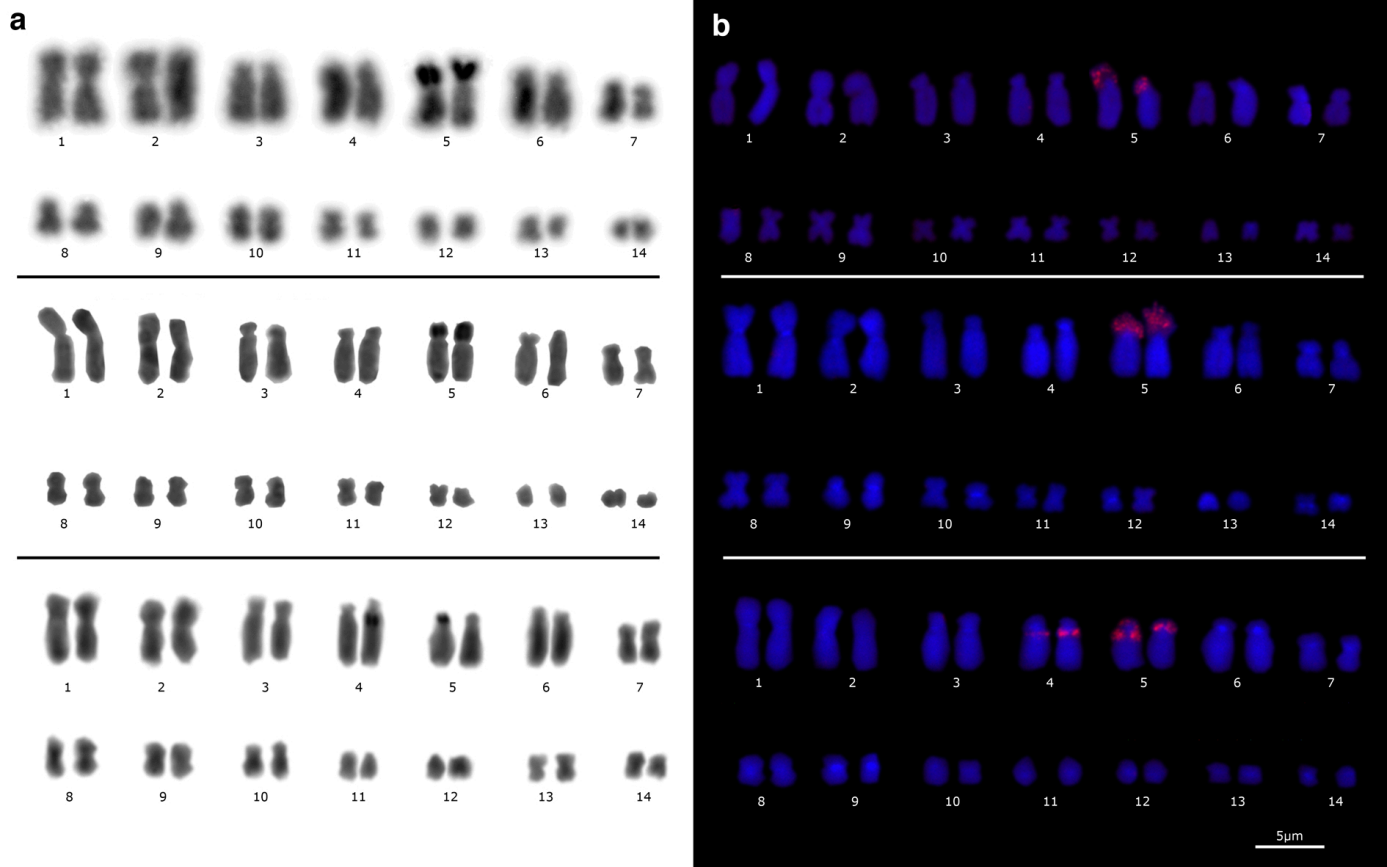
Ag-NOR staining (**a**), and FISH using 28S rDNA probe (**b**) on the *Eupsophus vertebralis* group mitotic plates. The *E. emiliopugini*, *E. vertebralis* and *E. emiliopugini* from Puyehue locality karyotypes are shown (top, middle, and bottom, respectively). Note colocalization of AgNOR and FISH signals on pair 5 (top and middle). FISH signals on four chromosomes, two of them AgNOR stained are observed in *E. emiliopugini* from Puyehue (bottom, see text for details).

Ag-NOR staining detected active NORs on short arms of chromosomes of pair 5 in both *E. emiliopugini* and *E. vertebralis* karyotypes (Fig. 4a, top and middle, respectively). This technique detected active NORs, corresponding to secondary constriction, on long arm from one chromosome of the pair 4, and on short arm from one chromosome of pair 5 (Fig. 4a, bottom) in the Puyehue specimen.

### Molecular cytogenetic techniques

Coincident with Ag-NOR staining results, signals on short arms of chromosomes of pair 5 in both *E. emiliopugini* and *E. vertebralis*, were detected through FISH using 28S rDNA probe (Fig. 4b, top and middle, respectively). In the Puyehue specimen, this probe detected a long arm region of chromosomes in pair 4 and short arm regions of chromosomes in pair 5 (Fig. 4b, bottom).

**Figure 5.**
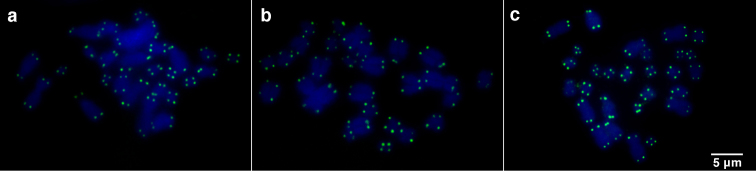
Fluorescent *in situ* hybridization over mitotic plates from the *Eupsophus vertebralis* group, using the telomeric probe. *Eupsophus emiliopugini* (**a**), *E. vertebralis* (**b**), and *E. emiliopugini* from Puyehue locality (**c**) mitotic plates are shown. Note the absence of interstitial signals in all chromosomes.

Telomeric, but no centromeric or interstitial signals were detected on all chromosomes in both species through FISH using universal telomeric probe (Fig. 5a, b, respectively). This pattern was also observed on mitotic plates from the Puyehue specimen (Fig. 5c).

## Discussion

### Karyotypic patterns of *E. emiliopugini* and *E. vertebralis*

We present the first comparative cytogenetic study using classical and molecular cytogenetic techniques among specimens from different localities of *E. emiliopugini* and *E. vertebralis*. According with previous works (Formas 1989, 1991), *E. emiliopugini* and *E. vertebralis* exhibit 2n = 28, and FN = 56 and 54, respectively, derived of polymorphisms in pair 13 (Fig. 2). We did not detect sex chromosomes in the *E. vertebralis* group as it was observed by Formas (1991) (Fig. 2). Since, the lineage that gave origin to *E. vertebralis* and *E. emiliopugini* diverged early in the evolutionary history of *Eupsophus* (Suárez-Villota et al. 2018a), and sex chromosomes are detected in some species of the *E. roseus* group (*E. roseus*, *E. migueli*, *E. insularis*, and *E. septentrionalis*; Iturra and Veloso 1986, Cuevas and Formas 1996, Veloso et al. 2005), we agree with the notion that sex chromosomes correspond to an apomorphic condition in *Eupsophus* (Iturra and Veloso 1986, King 1991, Cuevas and Formas 1996, Veloso et al. 2005).

C-banding has been largely used in amphibians to compare karyotypes and to distinguish species with the same diploid number (Bogart 1970, Cuevas and Formas 2003, Nogueira et al. 2015, Sangpakdee et al. 2017, Targueta et al. 2018). Moreover, homogeneous C-banding patterns among related species has been associated with low genetic differentiation (Pellegrino et al. 1997, Lourenço et al. 1998, Bruschi et al. 2012) and enrichment of repetitive elements, characteristic of amphibian chromosomes (Schmid et al. 1978, Bruschi et al. 2012, Zlotina et al. 2017). Therefore, the absence of interspecific variations in heterochromatin banding reported in this study (Fig. 3), could be associated with the recent and low differentiation between *E. vertebralis* and *E. emiliopugini* as has been reported in divergence times estimates and mitogenomic analyses (Suárez-Villota et al. 2018a, b).

### Nucleolus organizer regions (NORs)

Ag-NOR banding combined with FISH using rDNA probes allow us to characterize the NORs in *E. emiliopugini* and *E. vertebralis* (Fig. 4). NORs locus correspond to rDNA coding for 18S rRNA, 5.8S rRNA and, 28S rRNA (Preuss and Pikaard 2007, McStay 2016). Thus, while Ag-NOR staining detects active NORs, FISH checks the total number of loci rDNA (Zaleśna et al. 2017). For both species of the *E. vertebralis* group, excluding the Puyehue specimen, we detected Ag-NOR signals on the short arms of pair 5 (Fig. 4a, top and middle), colocalized with the secondary constriction, and with 28S rDNA FISH signal (Fig. 4b, top and middle, red signal). Therefore, rDNA locus was transcriptionally active in both homologues of pair 5 for *E. emiliopugini* and *E. vertebralis*. Thus, it was not possible to determine a species-specific pattern relative to numbers and locations of NORs between both species. Consequently, NORs polymorphism is not a well indicative of species differentiation in this group as occur in some species of *Alsodes* Bell, 1843 [*A. pehuenche* Cei, 1976, *A. vanzolinii* (Donoso-Barros, 1974) and *A. verrucosus* (Philippi, 1902); Cuevas and Formas 2003]. However, different situation occurs in some species of the *E. roseus* group. For example, *E. contulmoensis* and *E. migueli* show specific Ag-NOR banding patterns (Veloso et al. 2005).

Intraspecific polymorphism in NORs was detected in the Puyehue specimen (Fig. 4a, b, bottom). We observed CMA_3_ positive banding and 28S FISH signals on pairs 4 and 5 (four NOR loci, Figs 3b, 4b, bottom), of which only one chromosome of each pair showed secondary constriction (Fig. 2, bottom, black arrows) and Ag-NOR positive signal (Fig. 4a, bottom). The absence of secondary constriction in one chromosome from one pair is a cytologic phenomenon known as differential amphiplasty (Navashin 1928, Pikaard 2000). This phenomenon could be a manifestation of rRNA gene dosage control, regulating the number of active rRNA genes according to the cellular demand, or an epigenetic phenomenon from interspecific hybrids where the expression of rRNA genes inherited from one progenitor are silenced (Pikaard 2000, Tucker et al. 2010). Thus, the four rRNA loci with nucleolar dominance detected in Puyehue specimen could be related with chromosomal rearrangements (Schweizer and Loidl 1987), mobiles NORs (Schmid et al. 2017) or hybrid origin (Pereyra et al. 2009), as it has been also associated to polymorphic NORs in other species.

### Hypothesis about the evolution of pair 13

C-banding and CMA_3_/DAPI stains results did not show a heterochromatic region in the short arms of metacentric pair 13 of *E. emiliopugini* (Fig. 3a, b, top). Moreover, telomeric probe hybridized over *E. emiliopugini* and *E. vertebralis* mitotic plates detected telomeric/subtelomeric signals but not interstitial signals (Fig. 5). Therefore, our data did not support the addition of heterochromatic segments in the telocentric pair of *E. vertebralis* and not show insights of inversions in the pair 13 of the *E. vertebralis* group. Since, these phenomena could be expected under hypothesis to explain the differentiation of pair 13 in this group (Formas 1991), we cannot refuse the proposed explanations. In this regard, telomeric sequences at telomeric/subtelomeric region are conserved in vertebrates (Meyne et al. 1989) whereas interstitial telomeric sequences could result from chromosomal rearrangements in animals (Ruiz-Herrera et al. 2002, Vitturi et al. 2002, Castiglia et al. 2006). Therefore, the pericentric inversion proposed by Formas (1991) to explain the differences in pair 13 between *E. emiliopugini* and *E. vertebralis* could be unlikely or it did not include the telomeric regions. Additionally, interstitial telomeric sequences could also be lost, as it has been reported in mammals (Rogatcheva et al. 2002, Castiglia et al. 2006). Thus, we cannot falsify the inversion hypothesis in pair 13 of the *E. vertebralis* group.

In conclusion, our analyses corroborate species-specific cytogenetic pattern differences between *E. emiliopugini* and *E. vertebralis* by detecting metacentric or telocentric pair 13 in populations of these species, respectively. Although, our results do not allow rejecting hypotheses of chromosomal rearrangements or heterochromatin addition in the origin of chromosomes of pair 13, a euchromatic pattern without interstitial telomeric sequences characterized these chromosomes. We reported an intraspecific polymorphism related to number, location, and activation of NORs for one specimen of *E. emiliopugini* from Puyehue locality. Chromosome rearrangements, hybridization event and transposition could be involved in the origin of this polymorphism. Future studies using probes from chromosome 13, more samples of *E. emiliopugini* from Puyehue locality, and molecular sequences analyses will allow a better understanding of the chromosomal evolution in the *E. vertebralis* group.

## Supplementary materials

Supplementary material 1 Table S1. *Eupsophus* specimens analyzed in the present study Data type: speciemens data Explanation note: Map number (Fig. 1), species, localities, coordinates, numbers of samples, and vouchers from herpetological collection of Instituto de Ciencias Marinas y Limnológicas (UACh), are shown. https://binary.pensoft.net/file/375507This dataset is made available under the Open Database License (http://opendatacommons.org/licenses/odbl/1.0/). The Open Database License (ODbL) is a license agreement intended to allow users to freely share, modify, and use this Dataset while maintaining this same freedom for others, provided that the original source and author(s) are credited.Camila A. Quercia, Elkin Y. Suárez-Villota, Fausto Foresti, José J. Nuñez

## References

[B1] Blotto BL Nuñez JJ Basso NG Úbeda CA Wheeler WC Faivovich J (2013) Phylogenetic relationships of a Patagonian frog radiation, the *Alsodes* + *Eupsophus* clade (Anura: Alsodidae), with comments on the supposed paraphyly of *Eupsophus*. Cladistics 29: 113–131. 10.1111/j.1096-0031.2012.00417.x 34814377

[B2] Bogart JP (1970) Systematic problems in the amphibian family Leptodactylidae (Anura) as indicated by karyotypic analysis. Cytogenetics 9: 369–383. 10.1159/000130106 5501394

[B3] Bruschi DP Busin CS Siqueira S Recco-Pimentel SM (2012) Cytogenetic analysis of two species in the *Phyllomedusa hypochondrialis* group (Anura, Hylidae). Hereditas 149: 34–40. 10.1111/j.1601-5223.2010.02236.x 22458439

[B4] Castiglia R Garagna S Merico V Oguge N Corti M (2006) Cytogenetics of a new cytotype of African Mus (subgenus Nannomys) minutoides (Rodentia, Muridae) from Kenya: C- and G- banding and distribution of (TTAGGG)_n_ telomeric sequences. Chromosome Research 14: 587–594. 10.1007/s10577-006-1054-5 16823620

[B5] Cuevas CC Formas JR (1996) Heteromorphic sex chromosomes in *Eupsophus insularis* (Amphibia: Anura: Leptodactylidae). Chromosome Research 4: 467–470. 10.1007/BF02265054 8889246

[B6] Cuevas CC Formas JR (2003) Cytogenetic analysis of four species of the genus *Alsodes* (Anura: Leptodactylidae) with comments about the karyological evolution of the genus. Hereditas 138: 138–147. 10.1034/j.1601-5223.2003.01677.x 12921166

[B7] Fernández R Barragán MJL Bullejos M Marchal JA Díaz De La Guardia R Sánchez A (2002) New C-band protocol by heat denaturation in the presence of formamide. Hereditas 137: 145–148. 10.1034/j.1601-5223.2002.01672.x 12627841

[B8] Ferreira DC Oliveira C Foresti F (2011) Chromosome mapping of retrotransposable elements *Rex1* and *Rex3* in three fish species in the subfamily Hypoptopomatinae (Teleostei, Siluriformes, Loricariidae). Cytogenetic and Genome Research 132: 64–70. 10.1159/000319620 20798486

[B9] Formas JR (1978) A new species of leptodactylid frog (*Eupsophus*) from the coastal range in Southern Chile. Studies on Neotropical Fauna and Environment 13: 1–9. 10.1080/01650527809360528

[B10] Formas JR (1989) A new species of *Eupsophus* (Amphibia: Anura: Leptodactylidae) from Southern Chile. Proceedings of the Biological Society of Washington 102: 568–576. http://biostor.org/reference/65743

[B11] Formas JR (1991) The karyotypes of the chilean frogs *Eupsophus emiliopugini* and *E. vertebralis* (Amphibia: Anura: Leptodactylidae). Proceedings of the Biological Society of Washington 104: 7–11. https://archive.org/details/biostor-85810

[B12] Formas JR Brieva L (1994) Advertisement calls and relationships of chilean frogs *Eupsophus contulmoensis* and *E. insularis* (Amphibia: Anura: Leptodactylidae). Proceedings of the Biological Society of Washington 107: 391–397. https://archive.org/details/biostor-81214

[B13] Formas JR Lacrampe S Brieva L (1992) Allozymic and morphological differentiation among three South American frogs, genus *Eupsophus* (*E. roseus*, *E. insularis*, *E. contulmoensis*). Comparative Biochemistry and Physiology B 102: 57–60. 10.1016/0305-0491(92)90272-S 1526134

[B14] Frost DR (2019) Amphibian species of the world: an online reference. Version 6.0. http://research.amnh.org/herpetology/amphibia/index.html [accessed 10. september. 2019]

[B15] Hillis DM Dixon MT (1991) Ribosomal DNA: molecular evolution and phylogenetic inference. The Quarterly Review of Biology 66: 411–453. 10.1086/417338 1784710

[B16] Howell WM Black DA (1980) Controlled silver-staining of nucleolus organizer regions with a protective colloidal developer: a 1-step method. Experientia 36: 1014–1015. 10.1007/BF01953855 6160049

[B17] Ibarra-Vidal H Ortiz JC Torres-Pérez F (2004) *Eupsophus septentrionalis* n. sp., nueva especie de Leptodactylidae (Amphibia) de Chile central. Boletín de la Sociedad Biológica de Concepción 75: 91–112. https://biblat.unam.mx/es/revista/boletin-de-la-sociedad-de-biologia-de-concepcion/articulo/eupsophus-septentrionalis-n-sp-nueva-especie-de-leptodactylidae-amphibia-de-chile-central

[B18] Ijdo JW Wells RA Baldini A Reeders ST (1991) Improved telomere detection using a telomere repeat probe (TTAGGG)_n_ generated by PCR. Nucleic Acids Research 19: 4780. 10.1093/nar/19.17.4780 PMC328734 1891373

[B19] Iturra P Veloso A (1986) Further evidence for early sex chromosome differentiation of Anuran species. Genetica 78: 25–31. 10.1007/BF00058671 3248710

[B20] King M (1991) Chapter 15: The evolution of heterochromatin in the amphibian genome. In: Green D Sessions S (Eds) Amphibian Cytogenetics and Evolution.Academic Press. United States, 359–391. 10.1016/B978-0-12-297880-7.50019-6

[B21] Lourenço LB Recco-Pimentel SM Cardoso AJ (1998) Polymorphism of the nucleolus organizer regions (NORs) in *Physalaemus petersi* (Amphibia, Anura, Leptodactylidae) detected by silver staining and fluorescence *in situ* hybridization. Chromosome Research 6: 621–628. 10.1023/A:1009253410553 10099875

[B22] McStay B (2016) Nucleolar organizer regions: genomic “dark matter” requiring illumination. Genes and Development 30: 1598–1610. 10.1101/gad.283838.116 27474438 PMC4973289

[B23] Meyne J Ratliff RL Moyzis RK (1989) Conservation of the human telomere sequence (TTAGGG)_n_ among vertebrates. Proceedings of the National Academy of Sciences of the United States of America 86: 7049–7053. 10.1073/pnas.86.18.7049 2780561 PMC297991

[B24] Morescalchi A (1980) Evolution and karyology of the amphibians. Bolletino di Zoologia 47: 113–126. 10.1080/11250008009438709

[B25] Navashin MS (1928) «Amphiplastie» – eine neue karyologische Erscheinung. – Verfahren des V Internationalen Kongresses Vererbungswissenschaft. Berlin; Leipzig, 2: 1148–1152.

[B26] Nogueira L Zanoni JB Solé M De Mello Affonso PR Siqueira S Sampaio I (2015) Cytogenetic studies in six species of *Scinax* (Anura, Hylidae) clade *Scinax ruber* from northern and northeastern Brazil. Genetics and Molecular Biology 38: 156–161. 10.1590/S1415-4757382220140280 26273218 PMC4530648

[B27] Nuñez JJ (2003) Taxonomía y sistemática de las ranas del género *Eupsophus* (Leptodactylidae). Doctoral dissertation, Valdivia, Chile: Facultad de Ciencias, Universidad Austral de Chile.

[B28] Nuñez JJ Rabanal FE Formas JR (2012) Description of a new species of *Eupsophus* (Amphibia: Neobatrachia) from the Valdivian Coastal range, Southern Chile: an integrative taxonomic approach. Zootaxa 68: 53–68. 10.11646/zootaxa.3305.1.3

[B29] Pellegrino CK Kasahara S Rodrigues M Yonenaga-Yassuda Y (1997) Pericentric inversion events in karyotypic distinction of Brazilian lizards of genus *Phyllopezus* (Squamata, Gekkonidae) detected by chromosomal banding patterns. Hereditas 127: 255–262. 10.1111/j.1601-5223.1997.t01-1-00255.x

[B30] Pereyra MO Marti DA Lescano JN Rosset SD Baldo D (2009) Natural interspecific hybridization in *Odontophrynus* (Anura: Cycloramphidae). Amphibian-Reptilia 30: 571–575. 10.1163/156853809789647149

[B31] Pieczarka JC Nagamachi CY Paes de Souza AC Milhomem SSR de Castro RR Nascimiento AL (2006) An adaptation of DAPI-banding to fishes chromosomes. Caryologia 59: 43–46. 10.1080/00087114.2006.10797897

[B32] Pikaard CS (2000) The epigenetics of nucleolar dominance. Trends in Genetics 16: 495–500. 10.1016/S0168-9525(00)02113-2 11074291

[B33] Preuss S Pikaard CS (2007) rRNA gene silencing and nucleolar dominance: insights into a chromosome-scale epigenetic on/off switch. Biochimica et Biophysica Acta – Gene Structure and Expression 1769: 383–392. 10.1016/j.bbaexp.2007.02.005 PMC2000449 17439825

[B34] Rogatcheva MB Ono T Sonta S Oda S Borodin PM (2002) Robertsonian metacentrics of the house musk shrew (*Suncus murinus*, Insectivora, Soricidae) lose the telomeric sequences in the centromeric area. Genes and Genetic Systems 75: 155–158. 10.1266/ggs.75.155 10984840

[B35] Ruiz-Herrera A García F Azzalin C Giulotto E Egozcue J Ponsà M Garcia M (2002) Distribution of intrachromosomal telomeric sequences (ITS) on *Macaca fascicularis* (Primates) chromosomes and their implication for chromosome evolution. Human Genetics 110: 578–586. 10.1007/s00439-002-0730-6 12107444

[B36] Sangpakdee W Phimphan S Tengjaroenkul B Pinthong K Neeratanaphan L Tanomtong A (2017) Cytogenetic study of three microhylid species (Anura, Microhylidae) from Thailand. Cytologia 82: 67–74. 10.1508/cytologia.82.67

[B37] Schmid M Feichtinger W Weimer R Mais C Bolaños F León P (1978) Chromosome banding in Amphibia. Chromosoma 66: 361–388. 10.1159/000133929 7835080

[B38] Schmid M Steinlein C Feichtinger W Nanda I (2017) Chromosome banding in Amphibia. XXXV. Highly mobile nucleolus organizing regions in *Craugastor fitzingeri* (Anura, Craugastoridae). Cytogenetic and Genome Research 152: 180–193. 10.1159/000481554 29059674

[B39] Schweizer D (1976) Reverse fluorescent chromosome banding with chromomycin and DAPI. Chromosoma 58: 307–324. 10.1007/BF00292840 137107

[B40] Schweizer D Loidl J (1987) A model for heterochromatin dispersion and the evolution of C-band patterns. In: Stahl A Luciani JM Vagner-Capodano AM (Eds) Chromosomes Today.Springer, Dordrecht, 61–74. 10.1007/978-94-010-9166-4_7

[B41] Suárez P Cardozo D Baldo D Pereyra MO Faivovich J Orrico VGD Catroli GF Grabiele M Bernarde PS Nagamachi CY Haddad CFB Pieczarka JC (2013) Chromosome evolution in Dendropsophini (Amphibia, Anura, Hylidae). Cytogenetic and Genome Research 141: 295–308. 10.1159/000354997 24107475

[B42] Suárez-Villota EY Quercia CA Díaz LM Vera-Sovier V Nuñez JJ (2018a) Speciation in a biodiversity hotspot: phylogenetic relationships, species delimitation, and divergence times of Patagonian ground frogs from the *Eupsophus roseus* group (Alsodidae). PLoS ONE 13: 1–19. 10.1371/journal.pone.0204968 PMC6292574 30543633

[B43] Suárez-Villota EY Quercia CA Nuñez JJ (2018b) Mitochondrial genomes of the South American frogs *Eupsophus vertebralis* and *E. emiliopugini* (Neobatrachia: Alsodidae) and their phylogenetic relationships. Journal of Genomics 6: 98–102. 10.7150/jgen.26122 29973959 PMC6030769

[B44] Targueta CP Guerra V Gambale PG Bastos RP Silva D de M e Telles MP de C (2018) Cytogenetics of two hylid frogs from Brazilian Cerrado. Genetics and Molecular Biology 41: 814–819. 10.1590/1678-4685-gmb-2017-0382 30508007 PMC6415605

[B45] Tucker S Vitins A Pikaard CS (2010) Nucleolar dominane and ribosomal RNA gene silencing. Current Opinion in Cell Biology 22: 351–356. 10.1016/j.ceb.2010.03.009 20392622 PMC2912983

[B46] Veloso A Celis-Diez LJ Guerrero CP Méndez AM Iturra P Simonetti AJ (2005) Description of a new *Eupsophus* species (Amphibia, Leptodactylidae) from the remnants of Maulino Forest central Chile. Herpetological Journal 15: 159–165. http://repositorio.uchile.cl/handle/2250/163890

[B47] Vitturi R Libertini A Armetta F Sparacino L Colomba MS (2002) Chromosome analysis and FISH mapping of ribosomal DNA (rDNA), telomeric (TTAGGG)_n_, and (GATA)_n_ repeats in the Leech *Haemopis sanguisuga* (L.) (Annelida: Hirudinea) 115: 189–194. 10.1023/A:1020165425392 12403173

[B48] Zaleśna A Florek M Rybacki M Ogielska M (2017) Variability of NOR patterns in European water frogs of different genome composition and ploidy level. Comparative Cytogenetics 11: 249–266. 10.3897/CompCytogen.v11i2.10804 28919963 PMC5596979

[B49] Zlotina A Dedukh D Krasikova A (2017) Amphibian and avian karyotype evolution: Insights from lampbrush chromosome studies. Genes 8: 311. 10.3390/genes8110311 PMC5704224 29117127

