# Supplementary material for: Comparative cytogenetics of the ground frogs Eupsophus emiliopugini Formas, 1989 and E. vertebralis Grandison, 1961 (Alsodidae) with comments on their inter- and intraspecific chromosome differentiation
Source: Comp Cytogenet. 2020 Jan 27;14(1):61–74. doi: 10.3897/CompCytogen.v14i1.46852 (PMC6997241; doi:10.3897/CompCytogen.v14i1.46852)
Supplement: Supplementary material 1 [file comparative_cytogenetics-14-061-s001.docx]

**Supplementary Material 1**

**Table 1.** *Eupsophus* specimens analyzed in the present study. Map number (Fig. 1), species, localities, coordinates, numbers of samples, and vouchers from herpetological collection of Instituto de Ciencias Marinas y Limnológicas (UACh), are shown.

| **Nº Map** | **Species** | **Localities** | **Coordinates** | **Number of Samples** | **Voucher** |
| --- | --- | --- | --- | --- | --- |
| 1 | *E. vertebralis* | Tolhuaca | -38.280968,  -71.707993 | 1 | ICMLH439 |
| 2 |  | Lago Pellaifa | -39.610365,  -71.454444 | 3 | ICMLH544, 545, 555 |
| 3 |  | Colegual Alto | -39.411666,  -73.111975 | 1 | ICMLH500 |
| 4 |  | Chanchan | -39.580000,  -73.223763 | 1 | ICMLH506 |
| 5 |  | Oncol | -39.698333,  -73.327222 | 1 | ICMLH495 |
| 6 |  | Llancahue | -39.839166,  -73.130000 | 2 | ICMLH509, 570 |
| 7 |  | Chamil | -40.010697,  -73.107963 | 2 | ICMLH557, 558 |
| 8 |  | Cordillera Pelada | -40.103333,  -73.454444 | 2 | ICMLH407, 421 |
| 9 |  | Reumen | -39.951944,  -72.903333 | 1 | ICMLH374 |
| 10 | *E. emiliopugini* | Los Mañios | -40.332556,  -72.325420 | 2 | ICMLH550, 551 |
| 11 |  | Pucatrihue | -40.576410,  -73.703042 | 1 | ICMLH414 |
| 12 |  | Puyehue | -40.772533,  -72.417025 | 1 | ICMLH499 |
| 13 |  | Cordillera del Sarao | -41.164166,  -73.727222 | 2 | ICMLH403, 404 |
| 14 |  | Parque Alerce Andino | -41.605710,  -72.654490 | 1 | ICMLH467 |
| 15 |  | Huinay | -42.353739,  -72.434066 | 2 | ICMLH303, 304 |
